# Advantage of Semaglutide: Comprehensive Analysis of Metabolic Impact of Semaglutide‐Treated and Pair‐Fed Rats

**DOI:** 10.1002/cph4.70083

**Published:** 2025-12-17

**Authors:** Suyeun Byun, Morgan R. Sotzen, Mya A. Knappenberger, Madison T. Bento, Mohammed Asker, Doris I. Olekanma, Karolina P. Skibicka

**Affiliations:** ^1^ Department of Nutritional Sciences Pennsylvania State University State College Pennsylvania USA; ^2^ Huck Institutes of Life Science Pennsylvania State University State College Pennsylvania USA; ^3^ Department of Physiology and Pharmacology, Hotchkiss Brain Institute University of Calgary Calgary Alberta Canada

**Keywords:** adiposity, diet‐induced obesity, glucagon‐like peptide 1, ingestive behavior, sex differences

## Abstract

Semaglutide (SEMA), a GLP‐1 receptor agonist, effectively reduces body weight. Yet its mechanisms of action remain incompletely understood. It is unclear whether SEMA promotes weight loss solely through reduced food intake or also through intake‐independent mechanisms, and whether these effects differ by sex. To address these questions, we used a pair‐feeding design in diet‐induced obese rats, comparing SEMA‐treated rats with both *ad libitum*‐fed controls and SEMA intake‐matched groups over 4‐week treatment. Analyses included sex‐stratified outcomes, depot‐specific brown and white adipose profiling, thermogenesis, locomotor activity, and circulating metabolic hormone measurements. SEMA reduced food intake of both hypercaloric, high‐fat/high‐sugar diet and chow and produced body weight loss beyond the effects of caloric restriction alone. SEMA also curbed the hunger hormone ghrelin. It reduced visceral adiposity and increased activity, albeit more potently in females compared to males. Across adipose depots SEMA promoted smaller adipocyte size, white adipose tissue browning, and enhanced sympathetic innervation, while these changes were largely absent in pair‐fed rats. SEMA rescued caloric restriction‐associated hypothermia and small reductions in circulating thyroid hormones; it also potentiated local thyroid input. SEMA induced sex‐dependent, depot‐specific adipose remodeling and sustained increases in locomotor activity independent of food intake. Our integrative approach provides new insight into SEMA's mechanisms and highlights the importance of evaluating sex as a biological variable in mechanistic studies of obesity therapies. Metabolic benefits of the SEMA treatment far outweighed those offered by comparable caloric restriction, indicating that its mechanism of action involves not only hypophagia but also adipose tissue remodeling and browning.

## Graphical abstract

Semaglutide, GLP‐1 receptor agonist, offers metabolic benefits that far outweigh those offered by comparable caloric restriction. Its mechanism of action involves hypophagia but also adipose tissue remodeling and browning. Semaglutide targeted thermogenesis via alternation in the thyroid axis and sympathetic input to BAT. It curbed the hunger hormone, ghrelin, and increased GIP.

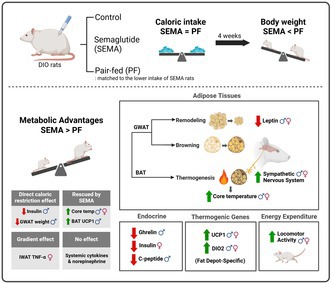

## 1 Introduction

Glucagon‐like peptide‐1 (GLP‐1) receptor agonists (GLP‐1RAs), including semaglutide (SEMA), have dramatically reshaped the therapeutic landscape for obesity, metabolic dysfunction, and type 2 diabetes. Large clinical trials have consistently demonstrated that SEMA induces substantial and sustained weight loss with accompanying improvements in glycemic control, cardiovascular risk, and obesity‐related complications including nonalcoholic steatohepatitis and hepatic fibrosis (Maretty et al. 2025; Sanyal et al. 2025; Reyes‐Garcia et al. 2025). These outcomes have led to expanding regulatory approvals and recommendations for GLP‐1RAs in obesity and related comorbidities.

Despite this clinical success, the precise mechanisms underlying its effects remain incompletely understood. GLP‐1 analogues are very effective at reducing appetite by targeting diverse central nervous system (CNS) GLP‐1 receptor (GLP‐1R) populations (Kanoski et al. 2016). They reduce not only ingestive behavior, but also food‐motivated behavior for palatable foods altogether, leading to significantly suppressed intake (Dickson et al. 2012; Skibicka 2013). While reduced food intake has long been considered the dominant driver of GLP‐1RA‐induced weight loss, this explanation might not fully capture the breadth of biological adaptations observed with GLP‐1 analogue treatment. This class of drugs has been shown to affect energy expenditure, yet results are not fully consistent. Some studies indicate reduced thermogenesis and locomotion after acute central or peripheral GLP‐1 or exendin‐4 administration in lean male rats (Hayes et al. 2008; Shirazi, Palsdottir, et al. 2013), or 2 or 3 days of exendin‐4 treatment in obese rats or mice (Goldberg et al. 2025; Blevins et al. 2024), presumably leading to reduced energy expenditure acutely, while others indicate increased brown adipose tissue thermogenesis‐related gene expression in lean or obese male mice after 5 days of brain administration of exendin‐4 or after acute brain liraglutide injection (Kooijman et al. 2015; Beiroa et al. 2014). Length of treatment, CNS‐site of drug application, species and specific agonists might have contributed to these discrepancies. It is also important to decouple the impact of GLP‐1analogues on thermogenesis from its effects on food—for example, there is normally a tight balance between intake and expenditure; thereby reduced food intake or fasting can result in reduced energy expenditure to restore energy balance. The long‐term, chronic impact of SEMA on thermogenesis, accounting for the SEMA‐generated caloric deficit, remains unknown. An equally important unanswered question is the relative contribution of SEMA‐induced weight loss over more direct effects of the drug on adipose tissue loss and remodeling.

Moreover, a comprehensive understanding of SEMA's impacts on circulating satiety or hunger hormones beyond reduced food intake is still lacking. Similarly, SEMA‐driven improvements in systemic inflammation have been suggested, but it remains unclear whether these benefits are simply a function of weight loss or perhaps weight loss‐independent direct effects of the drug.

Another critical but underexplored area regarding the mechanisms of action of SEMA is the potential sex differences (Borchers and Skibicka 2025; Jensterle et al. 2023). There are significant sex differences in metabolic and feeding responses to energy balance perturbations including fasting or diet‐induced obesity; thus it is reasonable to expect that anti‐obesity treatments might also show sex‐specific effects (Maric et al. 2022; Borchers et al. 2022; Asarian and Geary 2013; Arefin et al. 2025). The majority of preclinical GLP‐1 analogue studies excluded females, but emerging evidence indicates sex‐dependent GLP‐1 actions and potential interactions with estrogen signaling (Borchers and Skibicka 2025; Rentzeperi et al. 2022; Richard et al. 2016; Vogel et al. 2016), highlighting the need for sex‐stratified investigation of SEMA's mechanisms.

In addition, existing intake‐controlled studies typically focus on a single tissue or endpoint without looking at the comprehensive picture. As a result, there is limited understanding of how SEMA coordinates depot‐specific adipose remodeling with behavioral outcomes such as locomotor activity and endocrine adaptations. Because obesity reflects complex interactions among feeding behavior, energy expenditure, and tissue‐specific metabolism, addressing these gaps requires an approach that captures the interplay across systems.

Here, we sought to fill these gaps by employing a pair‐feeding design to isolate intake‐independent effects of SEMA, while simultaneously incorporating sex‐stratified analyses. SEMA treatment was applied to diet‐induced obese (DIO) rats, which were given a choice of chow and hypercaloric high‐fat/high‐sugar diet, to better model the choice aspect of the human diet. This integrated approach provides a more comprehensive understanding of how SEMA drives weight loss and metabolic remodeling beyond caloric restriction, and reveals sex‐dependent adaptations that may have translational relevance for precision treatment of obesity.

## 2 Materials and Methods

### 2.1 Animals

Male and female Sprague–Dawley rats (36 per sex; 7 weeks old upon arrival; Charles River, Wilmington, MA) were individually housed under a 12‐h light/dark cycle. During a one‐week acclimation period, rats had ad libitum access to standard chow (PicoLab Rodent Diet 5053; 3.37 kcal/g) and water. Following acclimation, the rats were additionally provided with a 60% high‐fat diet (HFD; D12462, Research Diets; 5.24 kcal/g) to induce DIO models, which was maintained for 6 weeks prior to treatment initiation and during treatment. The development of the DIO model was confirmed by comparing cumulative caloric intake and body weight gain with age‐matched rats maintained on standard chow from our laboratory. All procedures were approved by and conducted in accordance with the ethical guidelines of the Pennsylvania State University Institutional Animal Care and Use Committee (IACUC).

### 2.2 Surgery

One week before treatment began, the animals underwent subcutaneous implantation of telemetry devices (G2 E‐Mitter, Starr Life Sciences) for continuous monitoring of core body temperature and locomotor activity. Rats were anesthetized with an intraperitoneal injection of a ketamine (75 mg/kg) and xylazine (10 mg/kg) cocktail. For analgesia, meloxicam (2 mg/kg) was administered subcutaneously. Following aseptic preparation, a small midline abdominal incision (1–1.5 cm) was made, and the sterilized transmitter was inserted into the peritoneal cavity. The incision was closed in two layers using absorbable sutures for the muscle wall and wound clips for the skin. Animals were monitored daily until full recovery.

### 2.3 Drugs

Semaglutide (TLC Pharmaceutical Standards, USA) was dissolved in Milli‐Q water (MilliporeSigma) to prepare doses of 7 μg, 15 μg, 30 μg, 50 μg, and 70 μg, and stored at 4 C. Each dose was administered subcutaneously over consecutive two‐day intervals, to mimic the clinical protocol and minimize the potential malaise. Once the target dose of 70 μg was reached, it was maintained for the remaining 3 weeks of the intervention. This dose‐escalation (ramp‐up) protocol mimics clinical titration strategies designed to reduce gastrointestinal side effects and improve adherence (Cawthon et al. 2023). Injection volumes were calculated based on body weight (1 mL/kg). Control and pair‐fed animals received volume‐matched vehicle injections on the same schedule. All injections took place daily in the second half of the light cycle.

### 2.4 Pair‐Feeding Design

For each sex, rats were assigned to one of three groups: control (saline‐treated), SEMA‐treated (SEMA), or pair‐fed (PF; saline‐treated). Groups were equalized for body weight and food intake, to ensure lack of baseline differences between treatment groups within each sex. On day 0, SEMA animals began receiving subcutaneous SEMA injections according to the titration protocol. Beginning on day 1, PF animals were provided with the same total quantity of food (combined HFD and chow) that their SEMA‐matched counterparts had consumed the previous day. Each pair‐fed rat was individually yoked to a body weight‐matched SEMA‐treated rat. Pair‐fed rats received the same gram amount of each food (HFD and chow) consumed by their corresponding SEMA counterpart on the previous day. Food intake and body weights were monitored daily. To assess SEMA‐induced changes in food preference, both the control and SEMA groups had ad libitum access to two diet options (HFD and standard chow) throughout the study. In contrast, PF animals were provided with a fixed amount of food based on their matched SEMA pair's intake, without free access to the two diet types, to ensure intake of HFD and chow was matched to the SEMA group.

### 2.5 Core Temperature and Locomotor Activity

Core body temperature and gross motor activity were recorded using telemetry devices at different points in the study, with each cage placed on a platform receiver (ER‐4000 Energizer/Receivers; Starr Life Sciences). Data were collected and analyzed using VitalView Telemetry Software.

### 2.6 Brown Adipose Tissue Temperature

During week 3 of treatment, brown adipose tissue (BAT) temperature was assessed using thermal imaging. A 3 × 3 cm area between the shoulder blades was shaved 24 h before imaging to minimize stress‐induced changes in thermoregulation. Three thermal images per rat were captured from a fixed distance of 30 cm using an infrared camera (FLIR C5). BAT temperature was determined by averaging the mean temperature of the shaved area across the three images using FLIR Thermal Studio Suite software.

### 2.7 Tissue Collection

Following 4 weeks of intervention, animals were briefly anesthetized with isoflurane prior to euthanasia by decapitation for tissue and plasma collection. To ensure measured parameters are not reflective of most recent intakes all rats had food withheld for 2 h prior to euthanasia. Tissues were rapidly dissected, fresh‐frozen in isopentane chilled on dry ice, and stored at −80°C until analysis. Blood was collected immediately into two types of tubes: heparinized tubes for general plasma assays and P800 tubes (K2EDTA: anticoagulant with Protease, Esterase and DPP‐IV Inhibitors; Becton Dickinson) specifically for MSD metabolic hormone measurements. Plasma was separated by centrifugation and stored at −80°C until use.

### 2.8 Adipose Tissue Gene Expression

Total RNA was isolated from adipose depots (BAT, inguinal white adipose tissue (IWAT), and gonadal white adipose tissue (GWAT)) using the RNeasy Lipid Tissue Mini Kit (Qiagen) according to the manufacturer's protocol. RNA concentration and purity were assessed with a NanoDrop 2000 spectrophotometer (Thermo Fisher Scientific). Following the RNA isolation, cDNA was synthesized from 1000 ng of total RNA using the High‐Capacity cDNA Reverse Transcription Kit (Applied Biosystems, 4,368,814) in a 20 μL reaction volume. Quantitative PCR was performed with TaqMan Fast Advanced Master Mix (Applied Biosystems) on a QuantStudio 3 Real‐Time PCR System (Applied Biosystems). Gene expression was analyzed for inflammatory and thermogenic biomarkers. TaqMan Gene Expression Assays (Applied Biosystems) were used with the following probe IDs: UCP1 (Rn00562126_m1), DIO2 (Rn00581867_m1), PRDM16 (Rn01516224_m1), IL‐1β (Rn00580432_m1), IL‐6 (Rn01410330_m1), TNF‐α (Rn99999017_m1), MCP1 (Rn00580555_m1). All reactions were run in duplicate. Relative mRNA expression was calculated using the 2^‐ΔΔCt method, with beta‐actin (Actb; endogenous control) as the reference gene.

### 2.9 Plasma Hormone and Cytokine Quantification

Plasma concentrations of gut and metabolic hormones (GLP‐1, ghrelin, gastric inhibitory polypeptide (GIP), glucagon, insulin, C‐peptide, peptide YY (PYY), brain‐derived neurotrophic factor (BDNF), fibroblast growth factor 21 (FGF‐21), adiponectin, and resistin) and proinflammatory cytokines (interleukin‐1 beta (IL‐1β), interleukin‐6 (IL‐6), tumor necrosis factor‐alpha (TNF‐α), interferon‐gamma (IFN‐γ), keratinocyte‐derived chemokine/growth‐regulated oncogene (KC/GRO), interleukin‐4 (IL‐4), interleukin‐10 (IL‐10), and interleukin‐13 (IL‐13)) were measured using electrochemiluminescence immunoassays on the Meso Scale Discovery (MSD) platform (Meso Scale Diagnostics, Rockville, MD). Metabolic hormones were quantified with U‐Plex panels (Metabolic Combo 1 (rat); K15308K), adiponectin (K152YQR) and resistin (K152ZOR) with R‐Plex singleplex assays, and cytokines with the V‐Plex (Proinflammatory Panel 2; K15059D). Assays were performed according to the manufacturer's protocols, and plates were read using a QuickPlex SQ 120 instrument, and analyzed using MSD Discovery Workbench software.

In addition, circulating triiodothyronine (T3), thyroxine (T4), thyroid‐stimulating hormone (TSH), and norepinephrine (NE) were quantified using enzyme‐linked immunosorbent assay (ELISA) kits: Abcam Rat T3 ELISA (ab285258), Abcam Rat T4 ELISA (ab285259), Elabscience Rat TSH ELISA (EEL127), and Abcam Rat Norepinephrine ELISA (ab287789). Standards and samples were run in duplicate, and concentrations were calculated from standard curves generated for each analyte.

### 2.10 Histology and Adipocyte Size Analysis

IWAT and GWAT were collected, stored at −80°C, and subsequently embedded in OCT compound (Tissue‐Tek) for cryosectioning. Frozen blocks were sectioned at 10 μm using a cryostat and mounted on Superfrost Plus glass slides. Sections were air‐dried and stained with hematoxylin and eosin (H&E) using standard procedures. H&E‐stained sections were imaged at 10× magnification with a microscope. For each animal, three nonoverlapping fields were randomly selected from each tissue section. The cross‐sectional area (μm^2^) of individual adipocytes was measured using ImageJ software (NIH, Bethesda, MD) with the threshold‐based analysis. Cells were grouped into predefined size categories, and the average number of adipocytes per category was calculated. Data are expressed as the percentage of adipocytes within each size bin relative to the total number of adipocytes counted (Parlee et al. 2014).

### 2.11 Brown Adipose Tissue Immunohistochemistry

BAT tyrosine hydroxylase (TH) immunohistochemistry was performed to visualize sympathetic innervation (Mishra et al. 2019). BAT samples were embedded in OCT compound (Tissue‐Tek), frozen at −80°C, and cryosectioned at 10 μm using a cryostat. Sections were mounted on Superfrost Plus slides, air‐dried, and fixed in 4% paraformaldehyde (PFA) for 10 min at room temperature, followed by PBST (0.2% Triton X‐100 in PBS) permeabilization for 10 min at room temperature. Blocking was carried out for 1 h at room temperature in a solution prepared according to the secondary antibody host (normal donkey serum in 1% bovine serum albumin (BSA)). Sections were incubated overnight at 4°C with mouse monoclonal anti‐TH antibody (clone LNC1, MAB318, Millipore) diluted 1:150. Primary antibody was washed off using PBST, and then sections were incubated for 1 h at room temperature with donkey anti‐mouse IgG (H + L), Alexa Fluor 594 (Invitrogen) diluted 1:400. Slides were mounted with Vectashield Antifade Mounting Medium containing DAPI (Vector Laboratories). Images were acquired on a Leica DMi8 fluorescence microscope at 20× objective. Representative images were processed uniformly in ImageJ (NIH, Bethesda, MD) for brightness and contrast.

### 2.12 Statistical Analysis

Data are presented as mean ± SEM. Statistical significance was assessed using one‐ or two‐way ANOVA followed by Holm‐Sidak post hoc tests where appropriate (GraphPad Prism 10). A *p*‐value of < 0.05 was considered statistically significant.

## 3 Results

### 3.1 SEMA Reduces Food Intake and Promotes Weight Loss Without Compromising Muscle Mass

**FIGURE 1:**
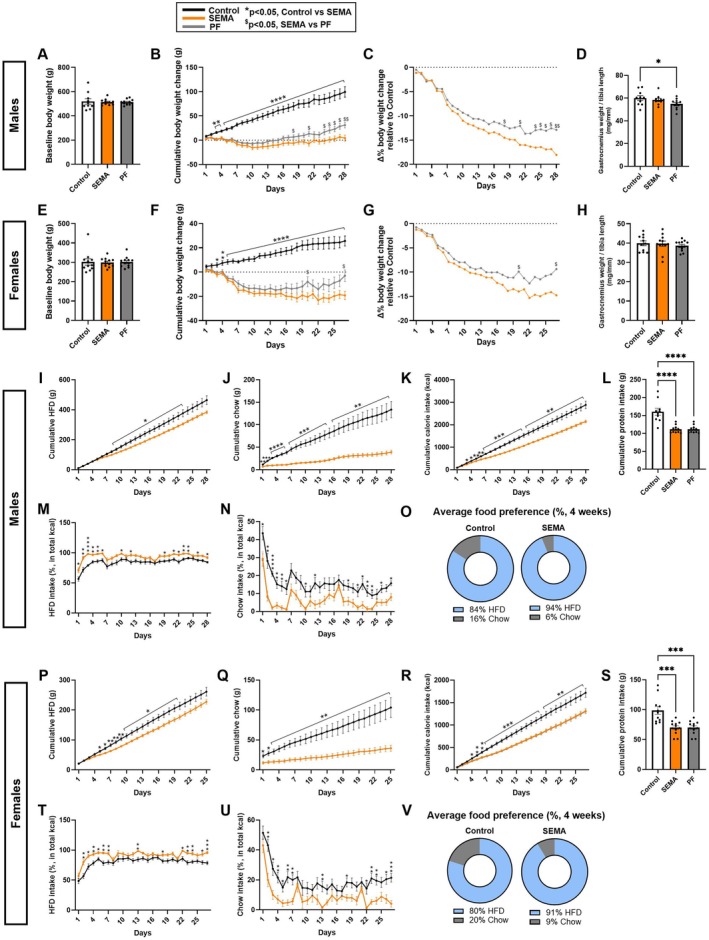
Effects of SEMA and pair‐feeding on body weight, muscle mass, and food intake in male and female rats. In males, baseline body weight was comparable across groups (A). SEMA prevented weight gain throughout treatment compared to control and PF (B). Body weight difference relative to control was significantly lower in SEMA‐treated rats compared with PF rats (C). Gastrocnemius mass, normalized to tibia length was preserved in SEMA, but reduced in PF rats (D). In females, baseline body weight did not differ between groups (E). SEMA induced a sustained weight loss below baseline, while PF rebounded toward baseline by the end of treatment (F). SEMA reduced body weight more than PF, relative to control (G). Muscle mass was preserved in both SEMA and PF female rats (H). In males, controls consumed more chow (I), HFD (J), total calories (K), and protein (L) than either SEMA or PF. SEMA shifted diet preference toward a higher proportion of HFD calories compared to control (M–O). In females, cumulative HFD (P), chow (Q), and total caloric intake (R) were reduced in both SEMA and PF versus controls, with parallel reductions in protein intake (S). Diet preference similarly showed higher proportional HFD intake in SEMA‐treated rats compared to controls (T–V). HFD, high‐fat diet; PF, pair‐feeding; SEMA, semaglutide. *N* = 10–12/group (males: 10–11; females: 11–12). Data are presented as mean ± SEM. *p < 0.05, **p < 0.01, ***p < 0.001, ****p < 0.0001. Line graphs: Control vs. SEMA (*p < 0.05, **p < 0.01, ***p < 0.001, ****p < 0.0001); SEMA vs. PF ($*p* < 0.05, $$*p* < 0.01, $$$*p* < 0.001, $$$$*p* < 0.0001).

Males. Baseline body weight was comparable across groups (Figure 1A). Daily body weight was monitored during treatment, and cumulative weight gain was analyzed. Two‐way ANOVA revealed significant effects of group (*F* [2, 29] = 50, *p* < 0.0001), time (*F* [1.41, 40.9] = 71.17, *p* < 0.0001), and their interaction (*F* (54, 783) = 29.96, *p* < 0.0001). Post hoc Holm‐Sidak tests revealed separation between control and SEMA groups emerged as early as day 3 and persisted throughout the study (Figure 1B). By the end of the study, at week 4, PF gained 31 ± 5.7 g, whereas SEMA‐treated rats remained close to baseline (4.3 ± 6.2 g), indicating a significant difference in weight gain between SEMA and PF groups. To better visualize change in body weight from baseline percent body weight change from control rats was calculated. Percent body weight change, relative to control, progressively diverged between SEMA and PF, reaching significance at week 3 (Figure 1C). To evaluate whether weight changes reflected altered muscle mass, gastrocnemius weight was normalized to tibia length (Figure 1D). Despite the large body weight difference, SEMA maintained muscle mass comparable to control, whereas PF displayed a reduced muscle index (*p* < 0.05). Intake of each diet (chow and HFD) and cumulative caloric intake were significantly higher in controls compared to SEMA and PF across most of the intervention (Figure 1I–K). Analysis of diet preference revealed group‐specific preferences: surprisingly the proportion of calories from HFD was higher in SEMA‐treated rats (group effect: *F* [2, 29] = 20.36, *p* < 0.0001; time effect: *F* [9.28, 269.1] = 23.26, *p* < 0.0001; interaction: *F* [54, 783] = 1.053, *p* > 0.05; Figure 1M), while chow intake was higher in controls (Figure 1N). On average across 4 weeks, SEMA consumed a greater proportion of total calories from HFD than controls (Figure 1O).

Females. Each group was balanced for baseline body weight (Figure 1E). SEMA‐treated rats consistently lost weight, ending the study below baseline (−19.3 ± 3.4 g; Figure 1F). PF animals, however, rebounded close to baseline by day 27 (−3 ± 4.8 g). Unlike males, neither SEMA nor PF female rats showed reductions in muscle mass relative to control (Figure 1H). Cumulative HFD intake (Figure 1P), chow intake (Figure 1Q), or total calories consumed (Figure 1R), and food preference (Figure 1T–V) showed a similar direction of effect as that observed in males.

Because decreased protein intake could influence muscle preservation, we calculated total protein intake from both diets. As expected from the overall reduced food intake, SEMA and PF rats consumed significantly less protein than controls in both males (*p* < 0.0001; Figure 1L) and females (*p* < 0.001; Figure 1S). Notably, despite this marked reduction, muscle mass was preserved in SEMA‐treated rats of both sexes.

### 3.2 SEMA Alters Systemic Metabolic Hormones Independent of Caloric Restriction

To assess how metabolic hormones were modulated by treatment independently of caloric restriction, we measured circulating plasma levels of these hormones at the conclusion of the treatment.

**FIGURE 2:**
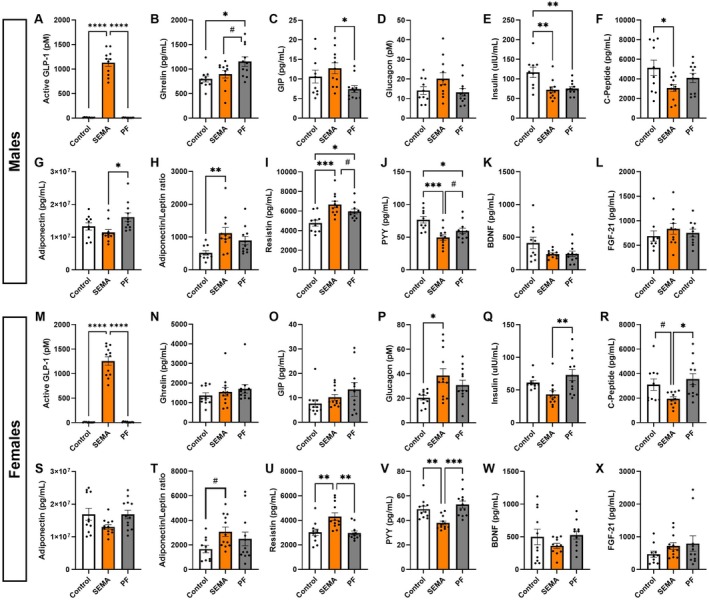
Plasma metabolic hormone concentrations in DIO male and female rats treated with SEMA or comparable caloric restriction. In males, SEMA markedly elevated circulating GLP‐1 compared to control and PF, confirming drug exposure (A). Ghrelin was increased in PF rats versus controls but blunted in SEMA‐treated rats, despite equivalent caloric restriction (B). GIP was elevated in SEMA compared to PF (C), while glucagon showed a nonsignificant trend toward higher levels in SEMA (D). Both SEMA and PF reduced insulin versus control (E). C‐peptide was decreased in SEMA‐treated rats versus control (F). Adiponectin was reduced in SEMA versus PF (G). Adiponectin‐to‐leptin ratio was increased in SEMA versus control (H). Resistin was elevated in both SEMA and PF relative to control (I). PYY was decreased in both groups, with the larger reduction in SEMA (J). No significant differences were observed for BDNF (K) or FGF‐21 (L). In females, GLP‐1 was significantly increased in SEMA relative to both control and PF (M). Ghrelin (N) and GIP (O) were unchanged. Glucagon was increased in SEMA versus control (P). Insulin (Q) and C‐peptide (R) were reduced in SEMA compared to PF, while PF showed no reduction relative to control. Adiponectin was not markedly different across groups (S). There was a trend toward increased adiponectin‐to‐leptin ratio in SEMA versus control (T). Resistin was selectively elevated in SEMA relative to both groups (U). PYY was significantly decreased only in SEMA, compared to both control and PF (V). BDNF (W) and FGF‐21 (X) remained unchanged. BDNF, brain‐derived neurotrophic factor; FGF‐21, fibroblast growth factor‐21; GIP, gastric inhibitory polypeptide; GLP‐1, glucagon‐like peptide‐1; PYY, peptide YY. *N* = 10–12/group (males: 10–11; females: 11–12). Data are presented as mean ± SEM. #*p* < 0.1, *p < 0.05, **p < 0.01, ***p < 0.001, ****p < 0.0001.

Males. GLP‐1 was measured in order to determine the levels of the circulating drug, as the exogenously delivered substance is likely to have overwhelmingly higher levels compared to the native GLP‐1; the concentration was markedly elevated in SEMA‐treated animals compared with other groups (*F* [2, 29] = 210, *p* < 0.0001; Figure 2A), confirming drug activity at the time of tissue collection. Ghrelin was increased in PF versus control (*p* < 0.05; Figure 2B), consistent with an energy deficit, whereas SEMA blunted this rise despite equivalent food restriction (vs PF: *p* = 0.06). GIP was elevated in SEMA relative to PF (*p* < 0.05; Figure 2C). Glucagon showed a nonsignificant trend toward higher levels in SEMA (*F* [2, 29] = 2.547, *p* = 0.09; Figure 2D). Both SEMA and PF rats had reduced insulin compared to control (all *p* < 0.01; Figure 2E), and C‐peptide was lower in SEMA (vs control: *p* < 0.05; Figure 2F), suggesting diminished glucose stimulation and improved insulin sensitivity. Although adiponectin levels were modestly reduced in SEMA compared to PF, the adiponectin‐to‐leptin ratio was significantly elevated in the SEMA‐treated rats compared to control (*p* < 0.01; Figure 2H), suggesting an overall improvement in adipose tissue function and systemic metabolic state (Figure 2G). Resistin was increased in both SEMA and PF rats, indicating an effect of caloric restriction (vs. control: *p* < 0.001; vs. PF: *p* = 0.09; Figure 2I). PYY was significantly reduced in both SEMA and PF, with the greater decrease in SEMA (vs control: p < 0.001, *p* < 0.05, respectively; Figure 2J). No group differences were detected for BDNF (Figure 2K) or FGF‐21 (Figure 2L).

Females. GLP‐1 was significantly elevated in SEMA‐treated animals compared with both control and PF groups (*F* [2, 32] = 226.4, *p* < 0.0001; Figure 2M), confirming drug activity at the time of tissue collection. Ghrelin (Figure 2N) and GIP (Figure 2O) were unchanged. Unlike the lack of effect in males, in females glucagon was significantly increased in SEMA versus control (*p* < 0.05; Figure 2P), suggesting compensatory regulation of glucose. Also contrary to what would be expected based on male data, insulin (Figure 2Q) and C‐peptide (Figure 2R) were both reduced in SEMA compared to PF (*p* < 0.01, *p* < 0.05, respectively), indicating enhanced insulin sensitivity in this group, but surprisingly PF female rats did not derive any benefit with respect to these hormones from food restriction. While adiponectin levels were not significantly different across groups (all *p* > 0.05; Figure 2S), the adiponectin‐to‐leptin ratio exhibited a trend toward increase in SEMA treated rats compared to controls (*p* = 0.095; Figure 2T), suggesting a shift toward an improved adipose tissue metabolic state. Similarly, only the SEMA group showed changes in resistin, which was significantly elevated in SEMA compared with both groups (all *p* < 0.01; Figure 2U). Also, PYY levels were significantly decreased in SEMA relative to control (*p* < 0.01) and PF (*p* < 0.001; Figure 2V) rats, potentially suggesting negative feedback on endogenous satiety signaling, but surprisingly only in the SEMA group. BDNF (Figure 2W) and FGF‐21 (Figure 2X) were unchanged.

### 3.3 Systemic Inflammation Remained Largely Unchanged by SEMA

**FIGURE 3:**
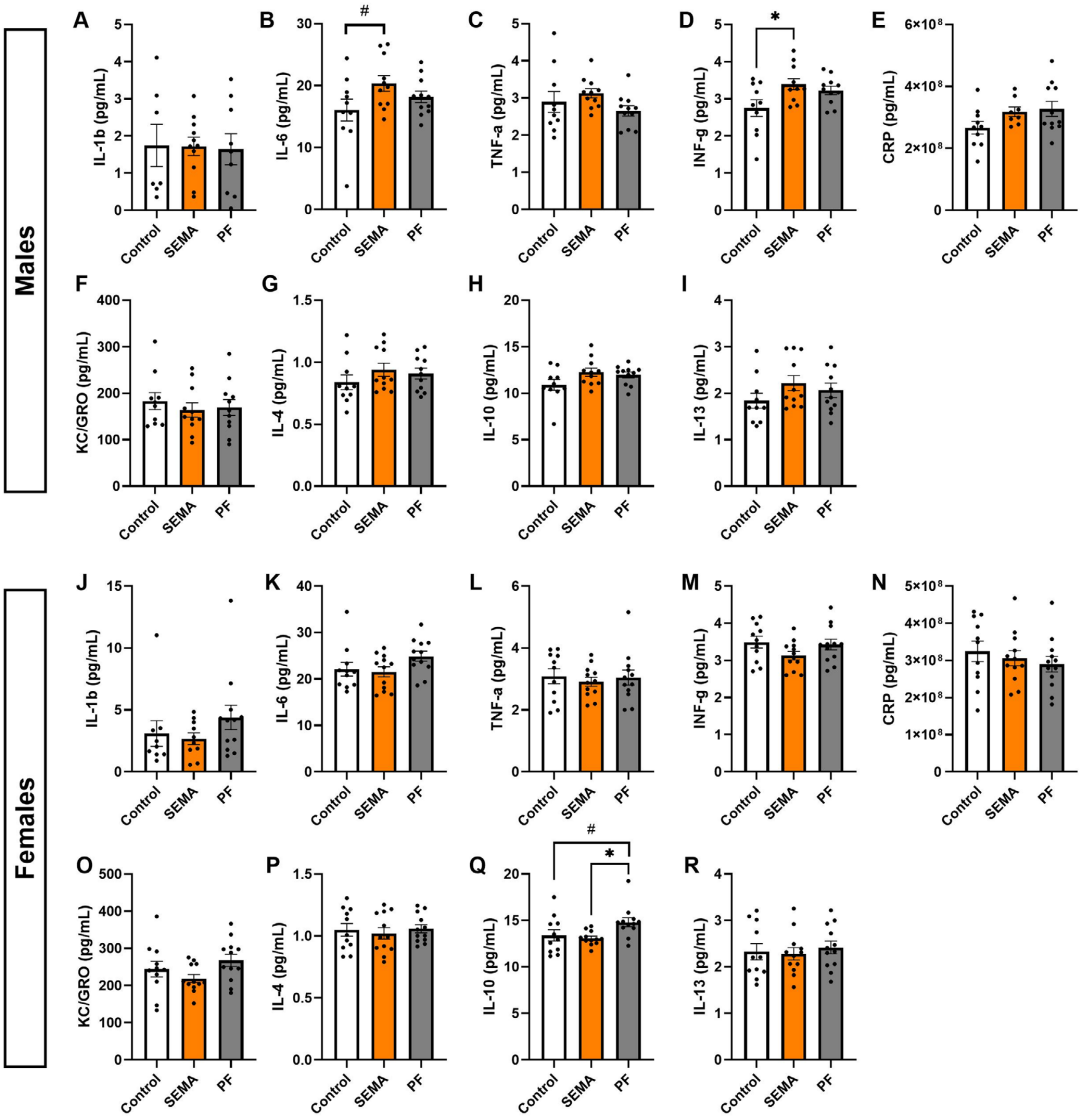
Plasma cytokine concentrations in DIO male and female rats treated with SEMA or comparable caloric restriction. In males, SEMA tended to increase IL‐6 versus control (B) and significantly increased IFN‐γ (D). Other cytokines IL‐1β (A), TNF‐α (C), CRP (E), KC/GRO (F), IL‐4 (G), IL‐10 (H), IL‐13 (I) showed no significant changes. In females, systemic cytokines were largely unchanged (J‐P, R), except for IL‐10 (Q), which was significantly reduced in SEMA compared to PF. CRP, C‐reactive protein; IFN‐γ, interferon‐gamma; IL‐10, interleukin‐10; IL‐13, interleukin‐13; IL‐1β, interleukin‐1 beta; IL‐4, interleukin‐4; IL‐6, interleukin‐6; KC/GRO, keratinocyte chemoattractant/growth‐related oncogene; TNF‐α, tumor necrosis factor‐alpha. *N* = 10–12/group (males: 10–11; females: 11–12). Data are presented as mean ± SEM. #*p* < 0.1, *p < 0.05.

Beyond metabolic outcomes, we examined whether SEMA modulates markers of systemic inflammation by measuring key circulating pro‐ and anti‐inflammatory cytokines. Contrary to our hypothesis, overall inflammatory status was largely unchanged in both sexes, with only a few cytokines showing treatment effects. In males, SEMA tended to increase IL‐6 compared to control (*p* = 0.09; Figure 3B), which is not surprising given the well‐established relationship between GLP‐1R activation and IL‐6 in males (Shirazi, Palsdottir, et al. 2013; Lopez‐Ferreras et al. 2018). It also significantly elevated IFN‐γ (*p* < 0.05; Figure 3D). In females, IL‐10 was significantly reduced in SEMA compared to PF (*p* < 0.05; Figure 3O).

### 3.4 SEMA Modulates GWAT Weight, Cell Size, and Local Cytokine Expression, Partly Independently of Caloric Restriction

To investigate how GWAT is modulated by SEMA, we assessed the depot weight, adipocyte size distribution, and inflammatory gene expression in this tissue.

**FIGURE 4:**
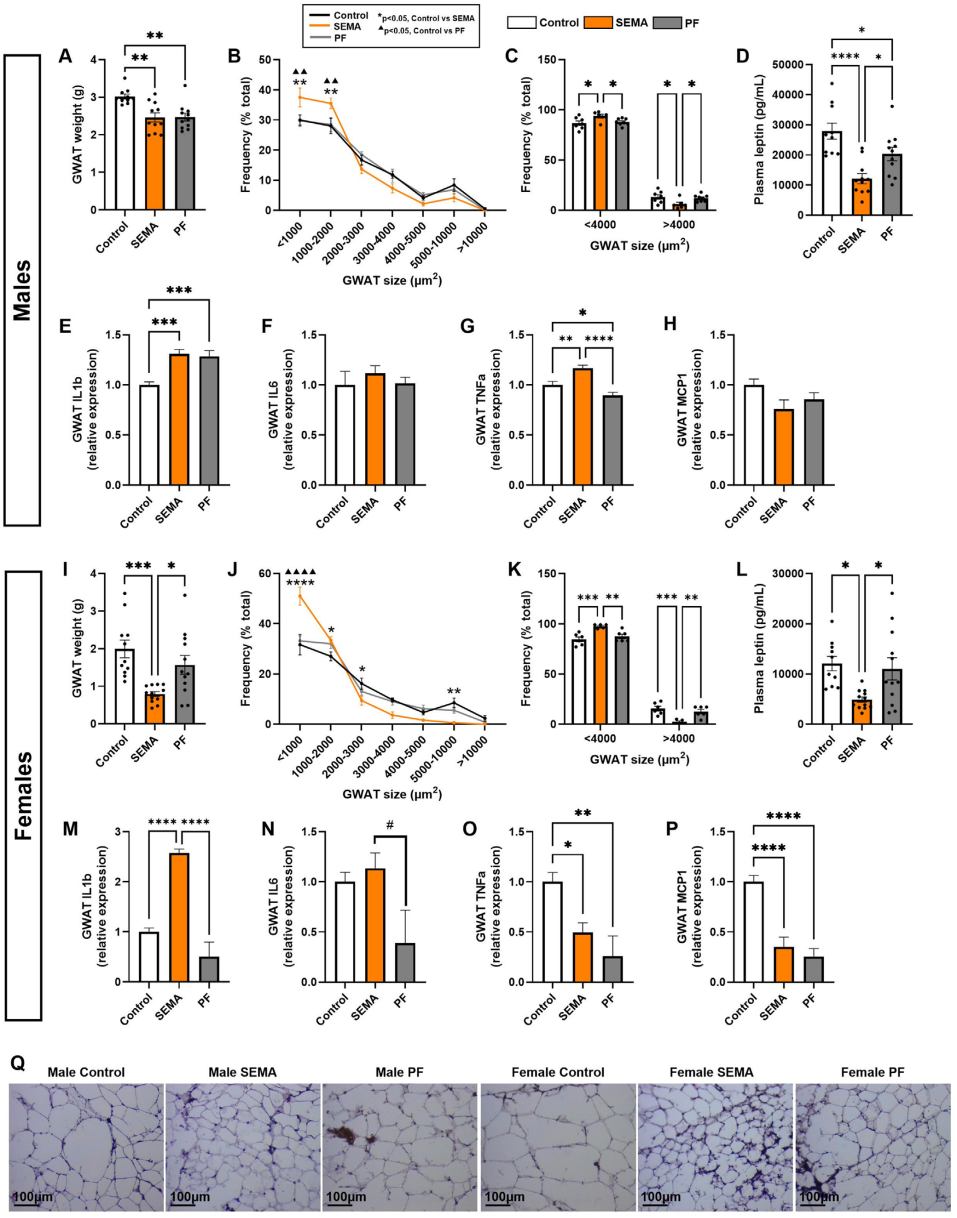
SEMA drives adipose tissue remodeling in DIO male and female rats, beyond that produced by comparable caloric restriction. In males, both SEMA and PF reduced GWAT mass compared to control (A). SEMA had a greater proportion of small adipocyte cells and fewer large cells (B, C) than control or PF. Plasma leptin was reduced in both groups, most strongly by SEMA (D). Local cytokines in male GWAT showed elevations of IL‐1β (E) and TNF‐α (G) in SEMA, while IL‐6 (F) and MCP‐1 (H) were unchanged. In females, only SEMA reduced GWAT mass compared to both PF and control (I). SEMA had more frequent smaller cells and less frequent larger cells (J, K). Plasma leptin was significantly reduced in SEMA (L). Inflammatory markers in female GWAT showed elevated IL‐1β in SEMA (M), a trend toward higher IL‐6 (N), but reduced TNF‐α (O) and MCP‐1 (P) in both SEMA and PF relative to control. Representative H&E‐stained IWAT sections for each group, shown separately by sex (Q). GWAT, gonadal white adipose tissue; H&E, hematoxylin and eosin; IL‐1β, interleukin‐1 beta; IL‐6, interleukin‐6; MCP‐1, monocyte chemoattractant protein‐1; TNF‐α, tumor necrosis factor‐alpha. *N* = 10–12/group (males: 10–11; females: 11–12). Data are presented as mean ± SEM. #*p* < 0.1, *p < 0.05, **p < 0.01, ***p < 0.001, ****p < 0.0001. Line graphs: Control vs. SEMA (*p < 0.05, **p < 0.01, **p < 0.0001); control vs. PF (^▲▲^ *p* < 0.01, ^▲▲▲▲^ *p* < 0.0001).

Males. Both SEMA and PF exhibited significantly reduced GWAT weight compared to control (all *p* < 0.01; Figure 4A), consistent with overall body weight loss. Although GWAT mass did not differ between SEMA and PF, adipocyte size distribution was distinct. Smaller adipocytes (< 2000 μm^2^) were more prevalent in SEMA (all *p* < 0.01; Figure 4B), while larger adipocytes (> 4000 μm^2^) were significantly less frequent compared to control and PF (all *p* < 0.05; Figure 4C). Supporting these morphological differences, circulating leptin was reduced by both PF and SEMA, but the reduction was most potent (over 60%) in the SEMA group (*p* < 0.05; Figure 4D). Despite the lack of systemic inflammatory changes, local cytokine levels in GWAT were altered. Both SEMA and PF showed elevated IL‐1β compared to control (all *p* < 0.001; Figure 4E). TNF‐α was also increased in SEMA compared to control (p < 0.01) and PF (*p* < 0.0001), while IL‐6 and MCP1 remained unchanged across groups.

Females. Surprisingly, and in contrast to male results, GWAT mass was not altered by the caloric restriction alone. SEMA exerted a pronounced effect on visceral fat in females, with GWAT weight significantly reduced compared to both PF (*p* < 0.05) and control (*p* < 0.001; Figure 4I), with GWAT mass was halved by SEMA. Adipocyte size distribution mirrored the male pattern, with smaller cells significantly more frequent and larger cells less frequent in SEMA relative to control (*p* < 0.001) and PF (*p* < 0.01; Figure 4J,K). In parallel, plasma leptin levels were markedly reduced in SEMA compared to other groups (all *p* < 0.05; Figure 4L). Local inflammatory changes were also observed. SEMA increased GWAT IL‐1β relative to both control and PF (all *p* < 0.0001; Figure 4M), indicating that in both sexes IL‐1β follows the measured GWAT weight loss pattern. A trend toward higher IL‐6 compared to PF (*p* = 0.06; Figure 4N) was detected. Interestingly, and in contrast to male GWAT results, caloric restriction reduced TNF‐α (SEMA: *p* < 0.05; PF: p < 0.01; Figure 4O) and MCP1 (all *p* < 0.0001; Figure 4P) compared to controls.

### 3.5 SEMA Reduced IWAT Mass With Limited Effects on Cell Size

Results obtained from the subcutaneous adipose depot, differed significantly from what we observed following semaglutide treatment in GWAT.

**FIGURE 5:**
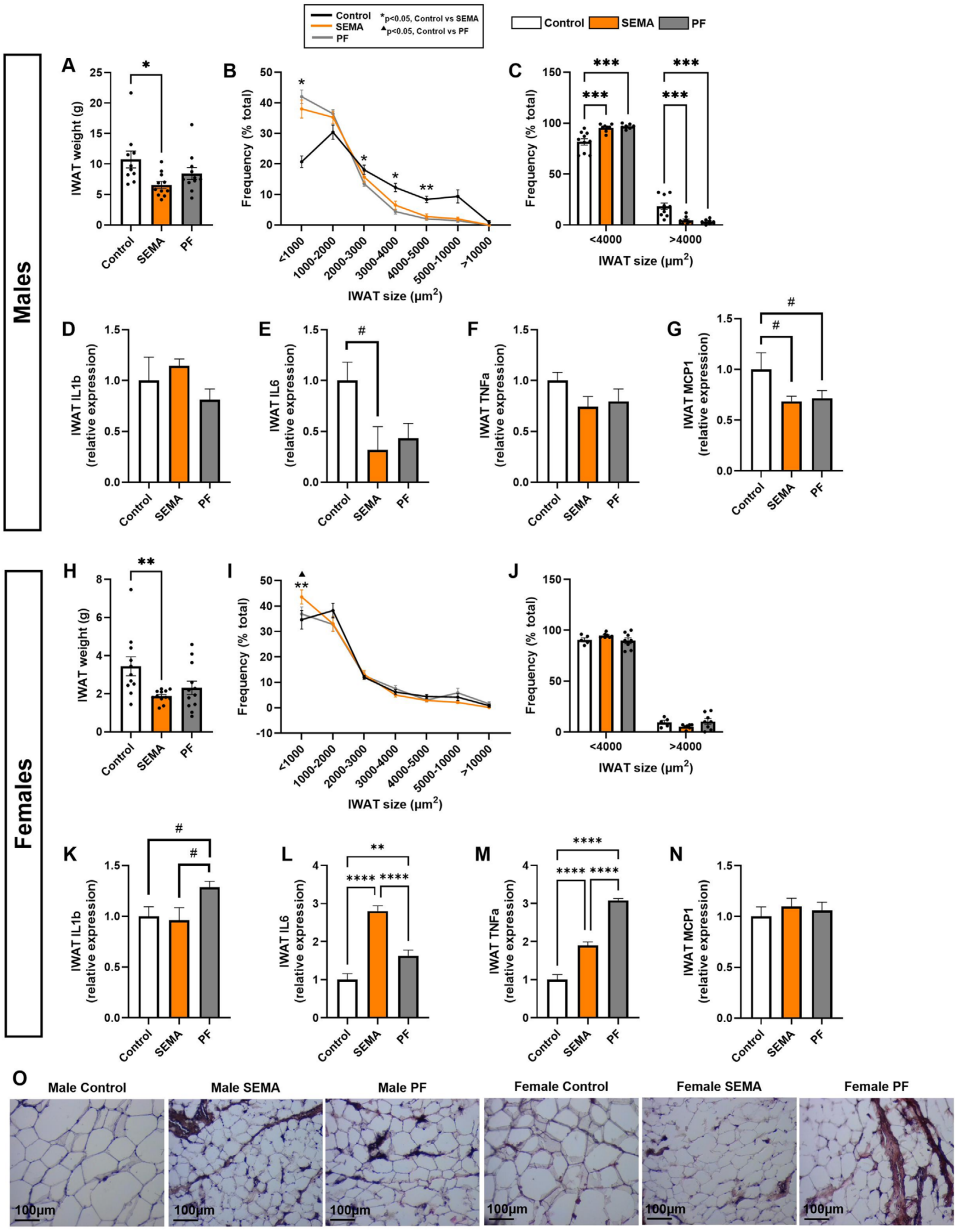
IWAT characteristics and inflammatory markers in male and female rats. In males, only SEMA reduced IWAT weight compared to control (A). Both SEMA and PF shifted adipocyte size distribution toward smaller cells (B, C). IWAT cytokines in males showed no group differences in IL‐1β (D) or TNF‐α (F), with nonsignificant trends toward lower IL‐6 (E) and MCP‐1 (G) in SEMA. In females, SEMA reduced IWAT weight compared to control (H), while PF preserved IWAT mass. Adipocyte size distribution did not differ across groups (I, J). Local cytokines diverged: PF showed a trend toward increased IL‐1β (K), whereas SEMA increased IL‐6 (L) and TNF‐α (M), though TNF‐α levels remained lower than in PF. MCP‐1 was unaffected (N). Representative H&E‐stained IWAT sections for each group, shown separately by sex (O). H&E, hematoxylin and eosin; IL‐1β, interleukin‐1 beta; IL‐6, interleukin‐6; IWAT, inguinal white adipose tissue; MCP‐1, monocyte chemoattractant protein‐1; TNF‐α, tumor necrosis factor‐alpha. *N* = 10–12/group (males: 10–11; females: 11–12). Data are presented as mean ± SEM. #*p* < 0.1, *p < 0.05, **p < 0.01, ***p < 0.001, ****p < 0.0001. Line graphs: Control vs. SEMA (*p < 0.05, **p < 0.01); control vs. PF (^▲^ *p* < 0.05).

Males. Only the SEMA group showed a significant reduction in IWAT weight compared to control (*p* < 0.05) at the end of treatment (Figure 5A). Yet, adipocyte size was markedly reduced, with a higher proportion of smaller cells and a lower proportion of larger cells relative to control (*p* < 0.001; Figure 5B,C) with similar contraction observed in PF, suggesting that this effect was primarily driven by reduced food intake. Subcutaneous adipose inflammation appeared less influenced by treatment, as IL‐1β (Figure 5D) and TNF‐α (Figure 5F) levels remained unchanged across groups. Nonetheless, a trend toward reduced IL‐6 (vs. control, *p* = 0.09; Figure 5E) and MCP1 (vs control, *p* = 0.08; Figure 5G) was noted in SEMA.

Females. Consistent with their significant body weight loss, the SEMA group displayed markedly reduced IWAT weight compared to control (*p* < 0.01; Figure 5H). Interestingly, under caloric restriction, PF rats preserved their IWAT mass, revealing an advantage of SEMA over caloric restriction for loss of this adipose depot mass. Treatment effects on IWAT morphology were modest, as the distribution of adipocyte sizes did not differ substantially between groups (Figure 5I,J). Inflammatory marker expression, however, diverged between groups. PF showed a trend toward increased IL‐1β (Figure 5K), whereas SEMA induced a robust increase in IL‐6 (*p* < 0.0001; Figure 5L) and elevated TNF‐α compared to control (*p* < 0.0001), though levels were significantly lower than in PF (*p* < 0.0001). IWAT MCP1 levels were unaffected by treatment.

### 3.6 SEMA Rescues the Food Restriction Driven Reduction in Body Temperature

Despite equal food consumption, SEMA groups weighed significantly less than PF groups by the end of the 4‐week intervention in both sexes. To explore the underlying energy‐expending mechanism, we measured core body temperature and tracked its changes over the course of treatment.

**FIGURE 6:**
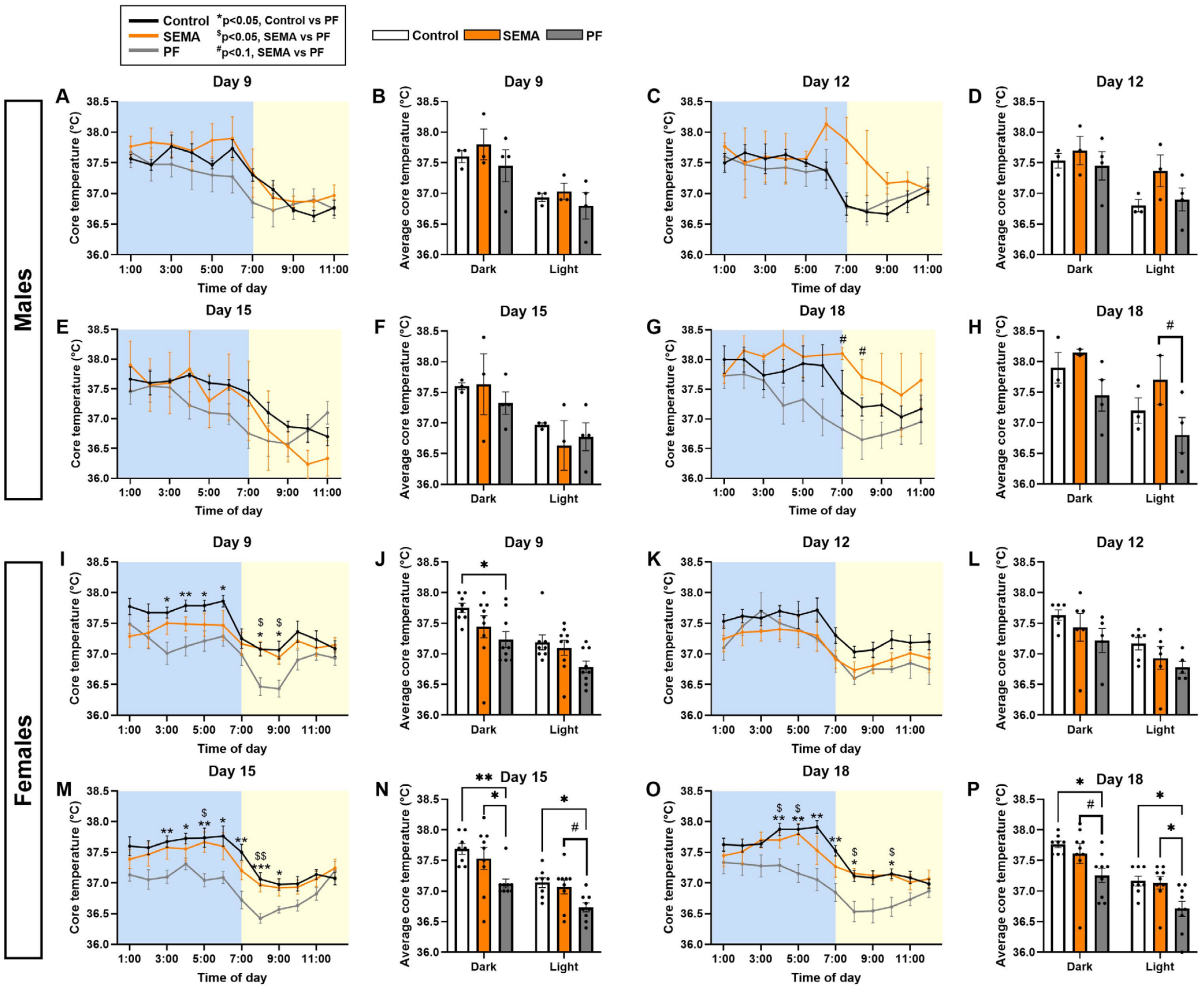
SEMA rescues caloric restriction‐induced hypothermia in male and female rats. In males, no differences in core body temperature were detected early (A–F). By day 18, SEMA showed higher temperature than PF, particularly in the light phase (G, H). In females, PF displayed reduced dark‐phase temperature by day 9 (I, J). Differences were absent at day 12 (K, L), but by day 15, SEMA rats showed higher dark‐phase temperature versus PF (M, N), with this hyperthermic effect persisting through day 18 (O, P). *N* = 3–4/group (males); *N* = 8–9/group (females). Data are presented as mean ± SEM. #*p* < 0.1, *p < 0.05, **p < 0.01. Line graphs: Control vs. PF (*p < 0.05, **p < 0.01, ***p < 0.001); SEMA vs. PF (#*p* < 0.1, $*p* < 0.05, $$*p* < 0.01).

Males. No significant differences in body temperature between groups were observed between groups until day 15 (Figure 6A–F). By day 18, however, SEMA showed a strong trend toward higher body temperature compared to PF rats during the early light phase (Figure 6G). When average core temperature was analyzed by dark/light phase (Figure 6H), two‐way ANOVA revealed a significant effect of time (*F* [1, 12] = 6.513, *p* < 0.05) and group (*F* [2, 12] = 3.935, *p* < 0.05), with no interaction. Post hoc analysis indicated a strong trend toward higher temperature in SEMA during the light phase compared to PF (*p* = 0.051).

Females. Temperature changes emerged earlier. By day 9 (Figure 6I,J), PF rats displayed significantly lower dark phase temperature compared to control (*p* < 0.05), potentially indicating that females more rapidly buffer the reduced caloric intake with reduced expenditure, at least as indicated by body temperature. Group differences became more pronounced by day 15 (Figure 6M,N), with SEMA rats showing significantly higher dark phase temperature relative to PF (*p* < 0.05) and a strong trend in the light phase (*p* = 0.06). This hyperthermic effect of SEMA persisted through the later stages of treatment (Figure 6O,P).

### 3.7 SEMA Preserves BAT Thermogenesis and Promotes Browning Pathways Under Caloric Restriction

As treatment induced changes in body temperature, we next investigated the underlying thermogenic mechanisms by examining tissues and genes involved in heat production.

**FIGURE 7:**
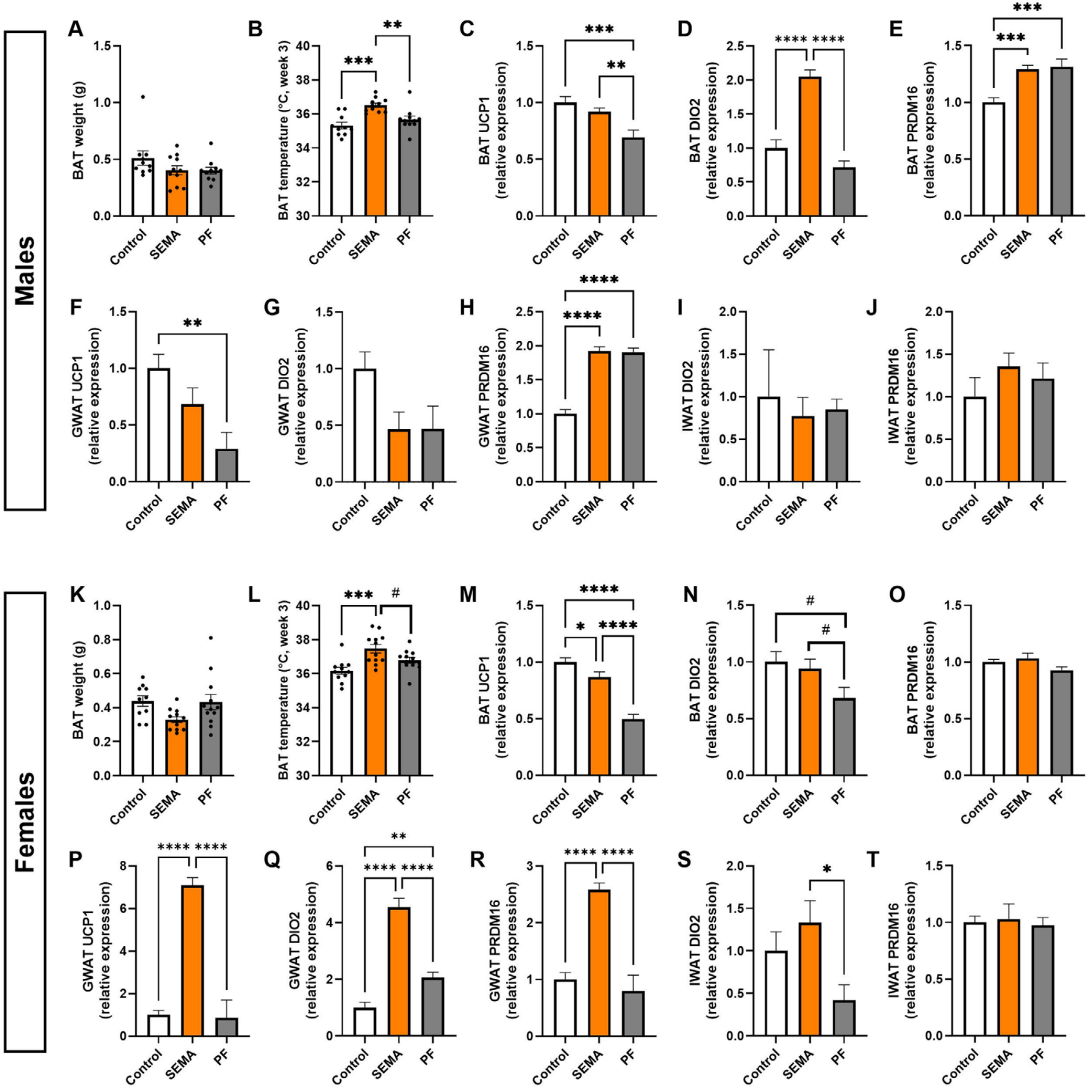
BAT, GWAT, and IWAT thermogenic capacity gene expression in male and female rats. In males, BAT weight was unchanged (A), but BAT temperature was elevated in SEMA (B). UCP1 expression was reduced in PF but preserved in SEMA (C). DIO2 was robustly increased in SEMA (D), and PRDM16 was elevated in both PF and SEMA (E). In male GWAT, UCP1 was reduced in PF but not SEMA (F), DIO2 was unchanged (G), and PRDM16 was increased in both groups (H). In male IWAT, DIO2 and PRDM16 were unchanged (I, J); UCP1 was undetectable. In females, BAT weight was unchanged (K), but BAT temperature (L) and UCP1 expression (M) were increased in SEMA. DIO2 was preserved in SEMA but reduced in PF (N), while PRDM16 was unaffected (O). In female GWAT, SEMA markedly upregulated UCP1 (P), DIO2 (Q), and PRDM16 (R). In female IWAT, DIO2 was elevated in SEMA compared to PF (S), while PRDM16 was unchanged (T); UCP1 was undetectable. BAT, brown adipose tissue; DIO2, type II iodothyronine deiodinase; GWAT, gonadal white adipose tissue; IWAT, inguinal white adipose tissue; PRDM16, PR domain containing 16; UCP1, uncoupling protein 1. *N* = 10–12/group (males: 10–11; females: 11–12). Data are presented as mean ± SEM. #*p* < 0.1, *p < 0.05, **p < 0.01, ***p < 0.001, ****p < 0.0001.

Males. Unlike other fat depots, BAT weight remained unchanged across groups (Figure 7A). Despite this, SEMA induced higher thermogenesis, as BAT temperature was significantly elevated (vs. control: *p* < 0.001; vs. PF: *p* < 0.01; Figure 7B). To probe potential thermogenic mechanisms, we analyzed thermogenesis‐related gene expression in BAT, GWAT, and IWAT. BAT UCP1 expression was markedly reduced only in PF rats (vs control: *p* < 0.001; vs. SEMA: *p* < 0.01; Figure 7C), consistent with the reduced core body temperature. In line with the BAT temperature results, SEMA robustly increased DIO2 levels (all *p* < 0.0001; Figure 7D), suggesting enhanced local thyroid hormone activation. PRDM16 expression was elevated in both caloric‐restricted groups (Figure 7E). In GWAT, UCP1 expression was decreased in PF compared to control (*p* < 0.01; Figure 7F), whereas no change was observed in SEMA, again consistent with the core body temperature results. DIO2 remained stable (Figure 7G), but PRDM16 expression was strongly upregulated in both PF and SEMA (vs control: all *p* < 0.0001; Figure 7H). IWAT was largely unaffected: DIO2 (Figure 7I) and PRDM16 (Figure 7J) were unchanged, and UCP1 was undetectable across groups (data not shown).

Females. As in males, BAT weight was unchanged, although BAT of SEMA treated rats tended to be lighter (vs control: *p* = 0.09; vs. PF: *p* = 0.09; Figure 7K). SEMA significantly elevated BAT temperature (vs control: *p* < 0.001; vs. PF: *p* = 0.056; Figure 7L). BAT UCP1 expression was robustly higher in SEMA compared to PF (*p* < 0.0001; Figure 7M), consistent with preserved thermogenesis under caloric restriction. PF showed a strong trend toward reduced DIO2 (vs control: *p* = 0.06; vs. SEMA: p = 0.09; Figure 7N), whereas SEMA prevented this decline, indicating preserved thyroid hormone signaling. BAT PRDM16 levels were unaffected (Figure 7O). In contrast to males, SEMA markedly upregulated UCP1, DIO2, and PRDM16 in GWAT (all *p* < 0.0001), aligning with the more pronounced core body hyperthermia observed in females. IWAT remained minimally affected: UCP1 was not detected (data not shown), PRDM16 levels were stable (Figure 7T), but DIO2 was upregulated in SEMA compared to PF (*p* < 0.05; Figure 7S).

### 3.8 SEMA Enhanced Sympathetic BAT Stimulation Without Altering Circulating Thyroid Hormones

While we noted substantial increases in DIO2 mRNA expression in BAT and GWAT in both sexes, we also wanted to probe whether this effect on the thyroid axis is contained locally or whether circulating hormone levels are also affected, reflecting the highly debated action of GLP‐1 analogues on the thyroid itself or on the CNS outflow to this organ. Moreover, since thyroid input represents only one potential mechanism of increased BAT and GWAT thermogenesis, we also examined potential alterations in the sympathetic nervous system (SNS) inputs.

**FIGURE 8:**
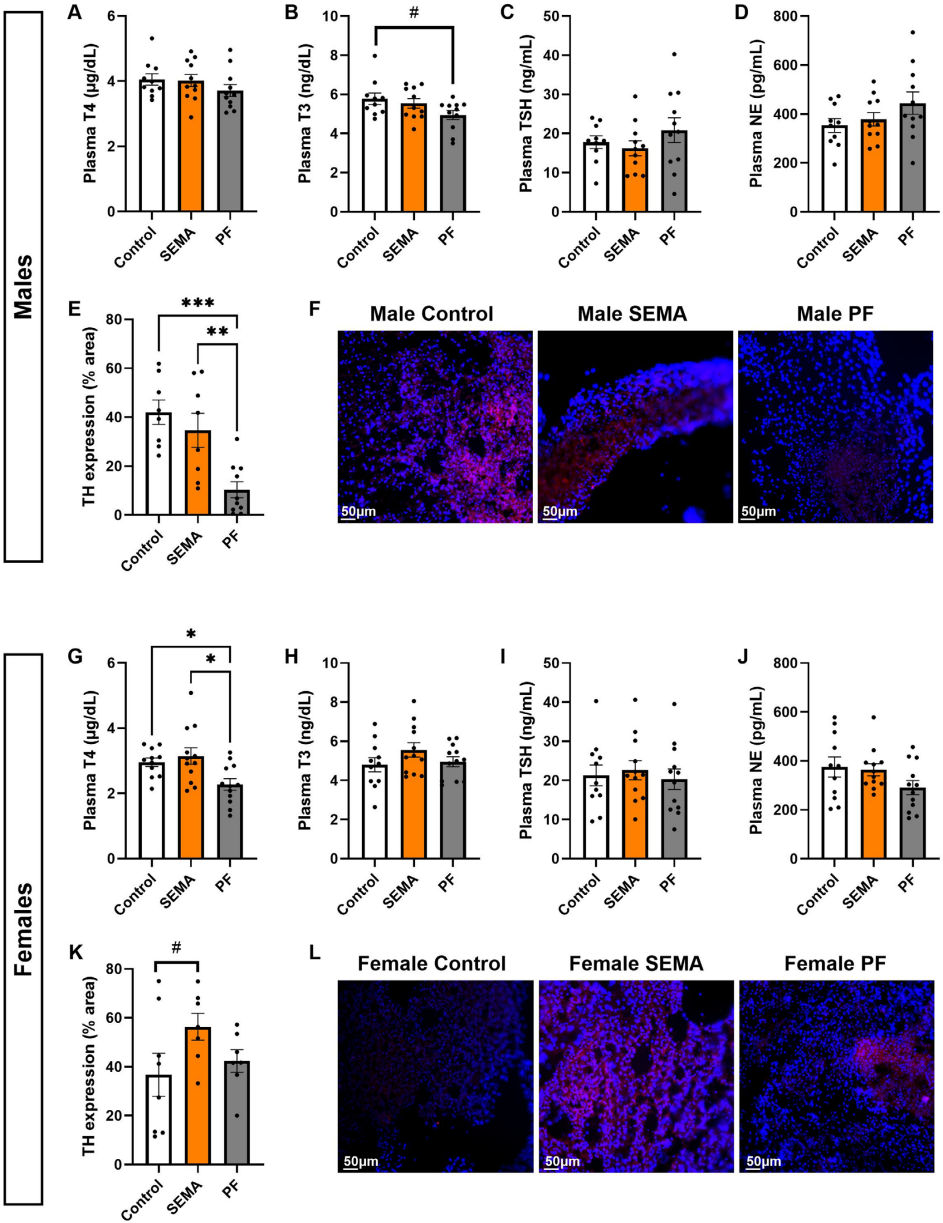
SEMA effect on thermogenic drivers: Thyroid axis and SNS output to BAT in male and female DIO rats. In males, plasma T4 (A), T3 (B), TSH (C), and NE (D) were unchanged. BAT TH‐positive fibers were elevated in SEMA compared to PF (E), indicating preserved sympathetic innervation. Male representative immunofluorescence images of TH expression (red) in BAT with DAPI nuclear staining (blue) by group (F). In females, plasma T4 was reduced by PF but preserved in SEMA (G). T3 (H), TSH (I), and NE (J) were unchanged. BAT TH expression showed a trend toward elevation in SEMA (K). Female representative immunofluorescence images of TH expression (red) in BAT with DAPI nuclear staining (blue) by group (L). BAT, brown adipose tissue; DAPI, 4′,6‐diamidino‐2‐phenylindole; NE, norepinephrine; T3, triiodothyronine; T4, thyroxine; TH, tyrosine hydroxylase; TSH, thyroid‐stimulating hormone. Sample sizes: Plasma measurements (males: *N* = 10–11, females: 11–12); BAT immunohistochemistry (males: *N* = 8–10/group; females: *N* = 7–8/group).

Males. Contrary to our hypothesis, circulating thyroid hormones (T3, T4) and TSH levels were not significantly altered (Figure 8A–C), although a trend toward reduction of T3 is consistent with the energy preservation and lower temperature in PF rats (vs control: *p* = 0.09). Norepinephrine (NE), a neurotransmitter central to thermogenic signaling, also showed no change in plasma levels (Figure 8D). However, SEMA markedly increased BAT tyrosine hydroxylase (TH)‐labeled fibers compared to PF (*p* < 0.01; Figure 8E), indicating preserved sympathetic stimulation of BAT and maintenance of UCP1 activation under caloric restriction in the SEMA group. Differences in TH innervation are illustrated in Figure 8F.

Females. Plasma T4 was reduced by feed restriction (all *p* < 0.05; Figure 8G) but preserved by SEMA. No changes were observed in T3, TSH, or NE (Figure 8H–J), suggesting that the drug's thermogenic effect was driven by adipose tissue adaptations in addition to alteration in available T4. A strong trend toward increased TH expression in SEMA (vs control: *p* = 0.0549; Figure 8K) further supports the notion that SEMA also enhances thermogenesis by augmenting NE‐driven BAT stimulation. Differences in TH innervation are illustrated in Figure 8L.

### 3.9 SEMA Enhances Locomotor Activity Independent of Reduced Intake, Contributing to Increased Energy Expenditure

**FIGURE 9:**
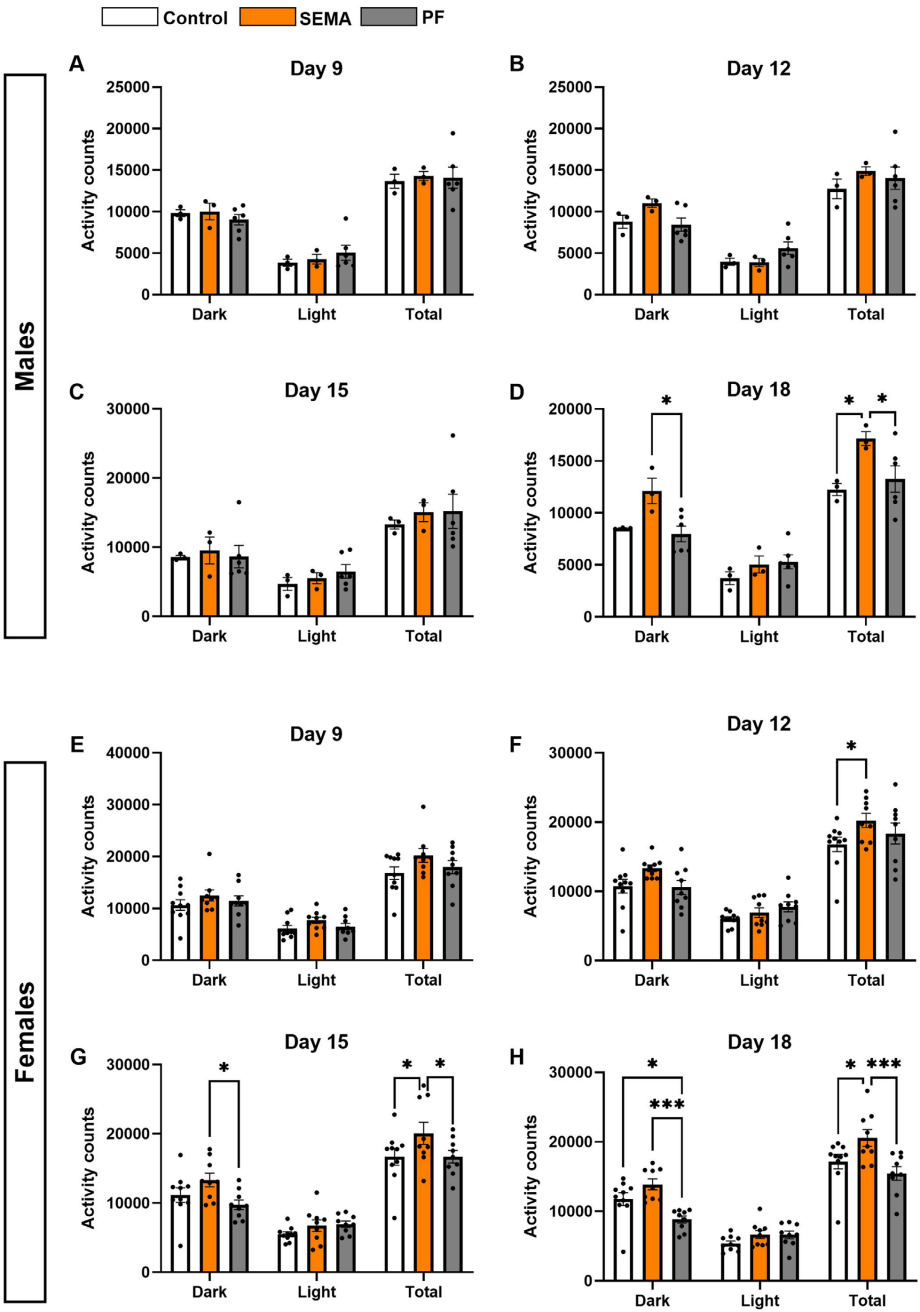
SEMA, but not equivalent caloric restriction, increases home cage locomotor activity in male and female DIO rats. In males, locomotor activity was similar across groups until day 15 (A–C). By day 18, SEMA increased dark‐phase and total activity compared to PF (D). In females, SEMA increased locomotor activity by day 12 (F), with elevations persisting at days 15 (G) and 18 (H); this effect was stronger during the dark phase. *N* = 3–4/group (males); *N* = 8–9/group (females). Data are presented as mean ± SEM. *p < 0.05, ***p < 0.001.

To further evaluate the drug's impact on energy expenditure, we monitored spontaneous locomotor activity in the home cages of the rats. In males, activity levels remained comparable across groups until day 15 (Figure 9A–C). By day 18, however, SEMA exhibited increased activity during the dark phase (vs PF: *p* < 0.05), and total 24 h activity was significantly elevated compared to other groups (all *p* < 0.05; Figure 9D). Elevated activity appeared earlier in females, with SEMA‐treated rats showing a significant increase in total locomotion by day 12 (vs control: *p* < 0.05; Figure 9F). This hyperactivity persisted at days 15 (Figure 9G) and 18 (Figure 9H), and was more pronounced in the dark phase than in the light phase.

## 4 Discussion

Our study demonstrates that chronic SEMA treatment in DIO male and female rats produces robust weight loss, distinguishing its effects from those of caloric restriction alone. Under conditions of equal food intake, SEMA‐treated animals exhibited persistent body weight reduction, and distinct adaptations in adipose, hormonal, and thermogenic systems. These findings indicate mechanisms of action beyond simple caloric restriction, emphasizing the drug's capacity to remodel adipose tissue, sustain thermogenesis, and enhance locomotor activity. Overall findings here can be grouped by those representing a direct result of reduced food intake (e.g., insulin levels in males, or GWAT weight in males), an effect present under caloric restriction/pair‐feeding but rescued by SEMA (e.g., core body temperature or BAT UCP‐1 in males), a gradient of effect (e.g., IWAT TNF‐α in females), or no effect of either treatment (e.g., most systemic circulating cytokines or plasma NE levels). All variations were detected, representing the need for tissue‐specific examination of effects, and a diverse mode of action of GLP‐1 analogues. Moreover, the variation detected differed by sex in many instances. The sex‐specific effects we identified, particularly greater visceral fat loss and earlier onset of body temperature preservation in females, reveal novel biological aspects of SEMA's therapeutic action.

### 4.1 Muscle Mass Impact

Despite substantial reductions in caloric and protein intake, SEMA preserved skeletal muscle mass, whereas pair‐feeding reduced muscle index specifically in males. This finding is notable because dietary restriction typically leads to loss of lean mass, with 20%–30% of weight loss in humans often derived from fat‐free tissue (Chaston et al. 2007; Weinheimer et al. 2010). Consistent with our findings, clinical data show that GLP‐1RAs promote preferential fat loss while sparing lean tissue, thereby preserving muscle to a greater degree relative to total weight loss than is typically achieved through diet alone (Wilding et al. 2021; Rodríguez Jiménez et al. 2024; Bikou et al. 2024) These results suggest that GLP‐1RAs may engage protective mechanisms that counteract the catabolic effects of protein restriction, allowing fat loss to occur without significant compromise of skeletal muscle. However, we would also approach these results with some caution, as our measurement represents only one muscle group, and in mice recent reports suggest loss of muscle mass with GLP‐1 analogue treatment (Karasawa et al. 2025; Nunn et al. 2024). Moreover, significantly reduced protein intake indicated here would also favor at least some loss of muscle mass. Yet, SEMA‐treated rats also increased their physical activity, and this was selective to SEMA and not PF rats, which might explain muscle preservation. It was also notable that muscle mass loss after caloric restriction was not found in females. While we can only speculate on the underlying reason, especially that there is a paucity of preclinical studies investigating muscle physiology after caloric restriction in female rats, it is plausible that the significantly higher levels of locomotor activity in females compared to males, irrespective of treatment, contributed to muscle preservation and overcame the effects of restriction. Overall, these are exciting early findings that will benefit from follow‐up work assessing whole body composition and functional measures of muscle function like muscle strength tests.

### 4.2 High‐Fat/High‐Sugar Diet Preference

Beyond its impact on body composition, SEMA altered dietary preference by increasing the proportion of calories derived from HFD (91%–94%) relative to controls (80%–84%). This outcome contrasts with most prior findings, where GLP‐1RAs typically reduced intake of calorie‐dense, palatable foods. In rodent studies, central GLP‐1R activation suppressed consumption of highly palatable foods through mesolimbic dopamine signaling (Dickson et al. 2012; Alhadeff et al. 2012; Wang et al. 2015), and tirzepatide, a dual GLP‐1/GIP receptor agonist, has been shown to suppress intake of palatable high‐fat/high‐sucrose diets and increase preference for chow (Geisler et al. 2023). Consistent with these findings, clinical studies reported that GLP‐1RA treatment dampened cravings for fat‐ and sugar‐rich foods (Blundell et al. 2017; Gibbons et al. 2021; Farr et al. 2016). A recent long‐term rat study, however, observed higher intake of low‐concentration sucrose solution in SEMA‐treated animals compared with controls during the weight‐maintenance phase and no decrease in intake of higher more calorically dense concentrations of sucrose in chronically SEMA‐treated rats relative to vehicle‐treated controls (Cawthon et al. 2023). Together this suggests that the timing of treatment effects may be important. In our study, the preservation of high‐fat/high‐sugar diet preference despite reduced overall intake may reflect a compensatory shift toward fat as a dense energy source under conditions of increased thermogenesis and locomotor activity. These adaptive nutrient selection mechanisms may contribute to the preservation of muscle mass by meeting energy demands without necessitating excessive protein breakdown.

### 4.3 Endocrine Impact

Compared to PF animals, SEMA induced endocrine changes that cannot be explained by food restriction alone. In males, the SEMA level caloric deficit produced the expected rise in ghrelin, whereas SEMA blunted this response. This indicates part of the advantage of having GLP‐1 analogues on board while under caloric deficit, is the suppression of the hunger hormone, something that certainly makes maintaining a reduced‐calorie diet easier. Ghrelin functions not only as a peripheral hunger signal but also as a regulator of reward circuits, amplifying food craving (Zhang et al. 2020; Skibicka et al. 2012), alcohol intake (Jerlhag 2024), and drug‐seeking (Merritt et al. 2023). Thus, suppression of the ghrelin rise by SEMA suggests an important mechanism by which GLP‐1 analogues might curb hunger while reducing reward‐driven consumption. Supporting this notion, preclinical studies have shown that GLP‐1RAsnot only reduce food motivated behavior (Dickson et al. 2012; Skibicka 2013) but also decrease alcohol intake in rodents when administered centrally (Shirazi, Dickson, and Skibicka 2013) or systemically (Chuong et al. 2023). Interestingly, ghrelin levels were not altered by pair feeding in females; consequently there was no rescue of this effect by SEMA. We can only speculate on the underlying mechanisms, but it is possible that females overall preserve energy more efficiently as evidenced by earlier reductions in body temperature shown here and preserved visceral fat, despite the caloric deficit in PF rats. This also indicated a fundamentally different metabolic and endocrine response to caloric deficit in males versus females.

SEMA also altered glucagon and GIP in a sex‐specific manner, yet these changes did not translate into greater insulin secretion. In females, SEMA significantly elevated glucagon, whereas in males it rescued GIP that was otherwise reduced in PF rats. Both hormones are known to participate in intra‐islet crosstalk, with glucagon acting on beta‐cell GLP‐1Rs and glucagon receptors to enhance insulin release, and GIP promoting glucagon secretion from alpha‐cells (Drucker and Holst 2023). Nevertheless, insulin and C‐peptide levels were consistently reduced in SEMA‐treated animals, indicating that the systemic effects of SEMA—reduced glucose stimulation and enhanced insulin sensitivity—overrode potential intra‐islet compensatory mechanisms. The divergence between elevated glucagon/GIP and lowered insulin and C‐peptide therefore highlights the context‐ and sex‐dependent nature of incretin regulation, where GLP‐1RAs may exert variable effects on alpha‐cell function depending on metabolic states and disease conditions (Nakashima et al. 2018; Saikia et al. 2021; Liu 2024).

Another notable difference between SEMA and PF rats was observed in PYY. The reduction in PYY was robust, present in both sexes, and perhaps unexpected, as both GLP‐1 and PYY are normally released post‐prandially to reduce subsequent feeding (De Silva and Bloom 2012). One possible explanation is that chronic pharmacological GLP‐1R activation may trigger negative feedback on endogenous PYY secretion. Supporting this idea, recent work demonstrated that combining PYY analogues with SEMA enhanced weight loss in DIO male rats (Oertel et al. 2024) but also increased gastrointestinal side effects in humans (Wulff et al. 2025), with tolerability depending on dose and the specific GLP‐1RA used (Liu et al. 2025). Thus, the suppression of endogenous PYY in our study may represent a compensatory mechanism for protection against excessive anorexia. This also indicates that PYY escapes GLP‐1R effects and represents a good partner for combination therapies.

### 4.4 Changes in Plasma Adipokines

Changes in plasma adipokines were paradoxical: pair‐feeding increased adiponectin in male rats, but SEMA rescued this change, while resistin was increased by both conditions in males, albeit to a larger extent by SEMA, and only by SEMA in females. Clinical studies have generally shown that GLP‐1RAs increase adiponectin and reduce or exert minimal effects on resistin (Simental‐Mendía et al. 2021; Yaribeygi et al. 2021), and strong associations between resistin and inflammatory mediators have been reported (Johnson and Olefsky 2013; Reilly et al. 2005; Shetty et al. 2004). In addition, several rodent studies have demonstrated a causal role of resistin in the development of insulin resistance (Muse et al. 2004; Steppan et al. 2001). In contrast, our SEMA‐treated rats exhibited lower insulin and C‐peptide, indicative of improved insulin sensitivity, while systemic inflammation remained largely unchanged. However, the observed elevation in resistin may reflect adipose tissue remodeling and the inflammatory response associated with adipose size reduction and apoptosis (Choe et al. 2016). This seems logical as the sex‐specific pattern of resistin changes also follows GWAT weight change pattern in both sexes as well as GWAT IL‐1β levels. Moreover, the ratio of adiponectin to leptin might represent another important measure of metabolic health (Fruhbeck et al. 2018). This ratio decreases after an obesogenic diet challenge in mice and obesity in humans (Fruhbeck et al. 2018, 2019; Becerril et al. 2022). Here the adiponectin to leptin ratio was significantly improved by SEMA in males, with a similar trend found in females, supporting improvements in metabolic health.

### 4.5 Circulating Neurotrophic and Growth Factors

A growing body of evidence indicates that GLP‐1RAs exert neuroprotective effects through modulation of BDNF signaling (Reich and Holscher 2022). Exenatide has been shown to elevate BDNF in the hippocampus and cortex and improve cognitive performance in mice (Bomba et al. 2018), and liraglutide reduced depression‐ and anxiety‐like behaviors in ovariectomized rats in association with changes in hippocampal BDNF (Sağlam et al. 2022). In our study, however, systemic BDNF levels were unchanged, suggesting that central BDNF measurements may provide greater insight into the neurotropic actions of GLP‐1RAs.

We did not detect any changes in circulating FGF‐21 following SEMA treatment or pair‐feeding. This finding contrasts with a short‐term mouse study in which 2 days of liraglutide administration elevated plasma FGF‐21 compared with control and PF groups (Le et al. 2023). Importantly, liraglutide, but not SEMA, elevated hepatic FGF‐21 protein levels in male mice (Liu et al. 2022), suggesting analogue‐specific interaction with the FGF‐21 signal. Other preclinical work has shown that co‐administration of GLP‐1 and FGF‐21 for 4 weeks produced synergistic effects on weight loss and glucose regulation in diabetic mice, surpassing the efficacy of GLP‐1 alone (Gilroy et al. 2020). The absence of an FGF‐21 response in our model therefore suggests that SEMA's metabolic effects may occur independently of endogenous FGF‐21 induction, but also raises the possibility that targeting FGF‐21 pathways in combination with SEMA could further enhance efficacy.

### 4.6 Inflammation and Adipose Remodeling

Previous studies have reported that GLP‐1RAs exert anti‐inflammatory effects both systemically and within adipose tissues (Maretty et al. 2025; Mehdi et al. 2023; Lee et al. 2012; Wong et al. 2024). In contrast, our results revealed mixed effects in white adipose tissue (WAT) that were dependent on both depot and sex. Although several inflammatory signals were altered within WAT in the current study, these changes did not translate into broad systemic inflammatory differences. Nevertheless, adipose remodeling should be considered a potential contributor to the inflammation‐related signals altered by SEMA.

The ability of GLP‐1 analogues to aid remodeling of adipose tissue has been well documented in prior studies in humans or mice (Lee et al. 2012; Emont et al. 2025). In line with these observations, we found that SEMA exerted stronger effects on visceral compared with subcutaneous fat depots in both sexes. A recent study demonstrated that epididymal fat cell size was significantly reduced in SEMA‐treated male mice with metabolic syndrome (Estato et al. 2025), and earlier work showed that GLP‐1 treatment reduced adipocyte size in epididymal fat of ob/ob mice (Lee et al. 2012), highlighting the drug's capacity to directly influence adipocyte morphology. Supporting these findings, our data indicate that gonadal fat atrophy is specifically a SEMA‐driven outcome, as PF animals did not exhibit comparable cellular remodeling, and importantly, this effect was evident in both males and females. The robust reductions of plasma leptin in both sexes occurred in parallel with GWAT adipocyte remodeling, further supporting the interpretation that SEMA exerts qualitative improvements in adipose tissue function. Notably, divergence between SEMA and PF rats was evident in both males and females, with SEMA producing greater reductions in leptin. Although gonadal fat remodeling was observed in both sexes, the impact of SEMA was more striking in females. Notably, dietary restriction alone was insufficient to reverse visceral adipocyte hypertrophy in females, while males showed partial responsiveness. It is noteworthy that females did not derive benefits from caloric restriction, when considering GWAT weight, leptin improvements, or adipocyte size reduction. This is also in line with females engaging compensatory mechanisms to prevent weight loss for example, hypothermia, much earlier than males. These findings highlight a potential sex‐dependent advantage of SEMA on visceral fat loss. Our data strongly suggest that females may derive greater benefit from SEMA treatment compared with caloric restriction. Clinical and mechanistic studies have also highlighted sex differences in GLP‐1 analogue responses (Borchers and Skibicka 2025), in adipose tissue insulin sensitivity (Arner et al. 2024), and in real‐world weight loss outcomes, where women achieved larger reductions than men following GLP‐1RA initiation (Marassi et al. 2025). Taken together, our findings underscore the potential advantage of SEMA over caloric restriction alone to improve metabolic health by targeting visceral fat, particularly in women. This advantage may reflect depot‐ and sex‐specific GLP‐1R distribution within the adipose tissue, although further studies are required to clarify this mechanism.

### 4.7 Impact on Thermogenesis; Sympathetic and Thyroid Mechanisms

Energy restriction is typically accompanied by a reduction in core body temperature, reflecting an adaptive strategy to conserve energy during negative energy balance (Duffy et al. 1989; Cannon and Nedergaard 2004). Consistent with this, PF rats in our study exhibited a hypothermic response. By contrast, SEMA treatment preserved and even elevated core temperature over time, with this protective effect against hypothermia becoming more pronounced with chronic treatment. Notably, the effect was more evident in females, highlighting a sex‐specific dimension of SEMA's thermogenic efficacy. This is in line with prior evidence that females display greater BAT activity and stronger browning responses than males (Palmer and Clegg 2015; Kim et al. 2016). At the molecular level, SEMA robustly rescued UCP1 in both sexes and DIO2 expression in females in BAT compared to PF rats. In males a robust SEMA‐specific increase in DIO2 was found. These elevations were coupled with significantly higher BAT temperature, in both sexes. BAT thermogenesis specifically represents yet another SEMA‐specific advantage, as an increase in BAT thermogenesis was not found in PF rats. Our findings are in line with prior studies showing that chronic peripheral administration of liraglutide enhances BAT activity and oxygen consumption, with effects mediated indirectly via central GLP‐1Rs (Kooijman et al. 2015). Our findings extend this body of work by demonstrating that systemic SEMA not only activates BAT thermogenic pathways but also prevents the hypothermic adaptation to caloric restriction and does so in females.

Central GLP‐1R activation through intra‐cerebroventricular delivery of GLP‐1 analogues not only stimulates sympathetic outflow to BAT, thereby increasing UCP1 expression and thermogenesis (Lockie et al. 2012), but also recruits beige adipocytes in WAT (Beiroa et al. 2014), at least in male mice. Consistent with this, SEMA has been shown to induce browning of subcutaneous and epididymal WAT in obese male mice (Martins et al. 2022). Our study extends these observations by demonstrating a pronounced browning effect in female GWAT, which was absent in males. This finding underscores both depot‐ and sex‐specific dimensions of SEMA's thermogenic actions that were not captured in earlier studies. Notably, females are inherently more prone to WAT browning, particularly in gonadal depots, due to estrogen‐driven sympathetic innervation (Kim et al. 2016). Such a sex‐specific effect on GWAT browning may represent a novel mechanism contributing to enhanced energy balance and warrants further investigation.

We pursued mechanisms via which BAT thermogenesis might be affected, that is, inputs to BAT driving increased expression of the thermogenic program. We hounded both thyroid and sympathetic drivers. Although SEMA markedly increased DIO2 expression across adipose depots, circulating thyroid hormones (T3 and T4) and TSH were not robustly affected, yet in male SEMA rescued T3 reduction induced by pair‐feeding, and in females T4 was rescued by SEMA. This dissociation with low systemic but robust local impact suggests that DIO2 upregulation primarily enhances local thyroid hormone activation within adipose tissue rather than altering systemic endocrine balance. This subtle and refined modulation is beneficial clinically, as large systemic changes in hypothalamic–pituitary–thyroid (HPT) axis are not desired. Likewise, clinical studies reported that GLP‐1RAs did not elevate systemic thyroid hormones and were instead associated with modest reductions in TSH, often attributed to weight loss rather than direct HPT axis stimulation (Sencar et al. 2019; Tee et al. 2023). Furthermore, a meta‐analysis of randomized controlled trials indicated no significant increase in hyperthyroidism, hypothyroidism, or thyroiditis with GLP‐1RA therapy (Hu et al. 2022). Together, these findings suggest that GLP‐1RAs promote depot‐specific thyroid hormone and sympathetic actions to facilitate thermogenesis while preserving systemic homeostasis.

We also observed increased TH expression in response to semaglutide. TH is a marker of SNS fibers, in brown adipose tissue of both sexes, consistent with enhanced sympathetic drive, a well‐established regulator of BAT thermogenesis (Cannon and Nedergaard 2004; Bartelt and Heeren 2014). The pattern of this elevation was somewhat sex‐divergent, where in males SEMA simply rescued the massive TH fiber depletion resulting from caloric restriction, whereas in females a trend to elevated TH in the SEMA group was found. This indicates that while the response of SNS innervation to BAT from caloric restriction is sex‐divergent, GLP‐1 analogue increases TH BAT fibers in both sexes.

### 4.8 Locomotor Activity

Beyond thermogenesis, our study revealed that chronic SEMA treatment increased locomotor activity, contributing to the greater body weight divergence between SEMA and PF rats, but also potentially aiding muscle mass preservation in the SEMA group, despite weight loss overall and reduced protein intake. Prior studies show mixed effects of GLP‐1RAs on physical activity. In mouse studies, SEMA delivered transdermally elevated both metabolic rate and activity (Li et al. 2025), while Gabery et al. (2020) found weight loss without changes in locomotion, suggesting energy expenditure (EE) adaptations can arise primarily from nonactivity components. Moreover, recent findings suggest reduced motivation to exercise when measured as the motivation to use running wheels in male mice (Foscue Jlj et al. 2024). By comparison, our findings indicate that spontaneous locomotor activity is a key contributor to enhanced EE during chronic SEMA treatment. Yet these outcomes may vary with treatment duration, species, sex, form of physical activity measured, and baseline metabolic state.

### 4.9 Overall Conclusions

In summary, our results demonstrate that SEMA produces weight loss through mechanisms extending beyond caloric restriction, including buffering counteractive endocrine signals, adipose remodeling, sustained thermogenesis, and increased locomotor activity. The sex‐specific outcomes observed—such as greater visceral fat loss, earlier onset of hyperthermia, and depot‐selective browning in females—highlight novel biological aspects of SEMA efficacy. Some of the findings highlight why females might derive a greater advantage of GLP‐1 analogues compared to fasting, and explain why “dieting” or caloric restriction, even when dutifully applied, might quickly run into homeostatic brakes preventing weight and adipose loss more so in females than males. Some of our findings also put forth ideas of partner molecules that might be ideal to enhance the therapeutic effects of GLP‐1 analogues, like PYY, since they are reduced in the SEMA condition, and simply supplementing them might take advantage of these physiological changes. Future work should clarify whether depot‐ and sex‐specific adaptations arise from differences in GLP‐1R distribution or signaling and explore how these mechanisms could be exploited in combination with other therapeutic strategies. Overall, our findings reveal surprising and in some cases sex‐specific, therapeutic advantages of SEMA while underscoring the need for continued mechanistic investigation.

## Author Contributions

K.P.S. supervised the project. K.P.S. and S.B. designed the experiments. S.B., M.R.S., M.A.K., M.T.B., M.A., D.I.O. performed the experiments and analyzed data. S.B. prepared the graphs and figures. The initial manuscript draft was prepared by S.B. and K.P.S. M.R.S., M.A.K., M.T.B., M.A., D.I.O. revised and approved the manuscript.

## Funding

This work was supported by the National Institutes of Health, R01DK129321, T32GM154124.

## Conflicts of Interest

The authors declare no conflicts of interest.

## Data Availability

The data that support the findings of this study are available from the corresponding author upon reasonable request.
